# Checkpoint inhibitor-induced gastritis followed by delayed severe hepatitis in a patient with lung metastases of head and neck squamous cell carcinoma: a case report

**DOI:** 10.3389/fonc.2023.1164236

**Published:** 2023-05-12

**Authors:** Tomoyuki Otsuka, Yoshiko Hashii, Sei Murayama, Yasunobu Ishizuka, Yoshiki Kojitani, Minako Nishio, Toshihiro Kudo

**Affiliations:** ^1^ Department of Medical Oncology, Osaka International Cancer Institute, Osaka, Japan; ^2^ Department of Pediatrics, Osaka International Cancer Institute, Osaka, Japan

**Keywords:** gastritis, immune-related adverse events, head and neck squamous cell carcinoma, case report, delayed severe hepatitis

## Abstract

Pembrolizumab, an anti-programmed death-1 (PD-1) receptor monoclonal antibody, is an effective first-line therapy for metastatic head and neck squamous cell carcinoma. Immune-related adverse events (irAEs) are well-described complications of PD-1 inhibitors, and multiorgan irAEs are known to occur occasionally. We report a patient with pulmonary metastases of oropharyngeal squamous cell carcinoma (SCC), who developed gastritis followed by delayed severe hepatitis and recovered with triple immunosuppressant therapy. A 58-year-old Japanese male with pulmonary metastases of oropharyngeal SCC who was treated with pembrolizumab, subsequently developed new-onset appetite loss and upper abdominal pain. Upper gastrointestinal endoscopy revealed gastritis and immunohistochemistry revealed pembrolizumab-induced gastritis. The patient developed delayed severe hepatitis at 15 months after initiating pembrolizumab treatment, presenting “Grade 4 aspartate aminotransferase increase” and “Grade 4 alanine aminotransferase increase.” Impaired liver function persisted despite pulse corticosteroid therapy with intravenous methylprednisolone 1,000 mg/day, followed by oral prednisolone 2 mg/kg/day and oral mycophenolate mofetil 2,000 mg/day. Tacrolimus, which reached target serum trough concentrations of 8–10 ng/mL, gradually improved irAE grades from Grade 4 to Grade 1. The patient responded well to triple immunosuppressant therapy comprising prednisolone, mycophenolate mofetil, and tacrolimus. Therefore, this immunotherapeutic approach could be effective for multiorgan irAEs in patients with cancer.

## Introduction

Immune checkpoint inhibitor (ICI) therapy is currently a standard therapeutic option for patients with metastatic head and neck squamous cell carcinoma (1), and appropriate management of immune-related adverse events (irAEs) is mandatory to maximize its clinical benefits. Multiorgan irAEs have been reported for ICIs including atezolizumab, nivolumab, and pembrolizumab (2–4). On the other hand, the first case of nivolumab-induced gastritis as a single organ irAE was reported by Kobayashi et al. (5). To date, gastritis associated with ICI therapy remains poorly recognized owing to the paucity of clinical evidence. Herein, we report on a patient with pulmonary metastases of oropharyngeal squamous cell carcinoma (SCC) who developed multiorgan irAEs—pembrolizumab-induced gastritis followed by delayed severe hepatitis.

## Case presentation

**Figure 1:**
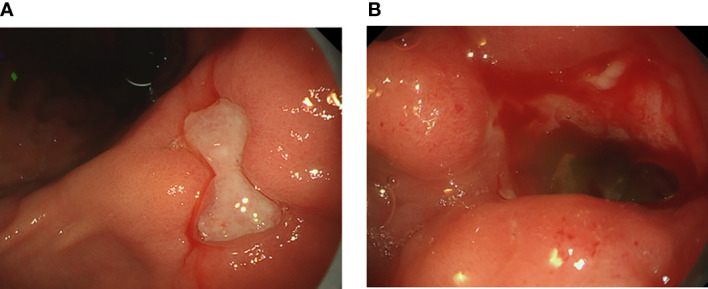
Upper gastrointestinal endoscopic images showing **(A)** a deep, large mucosal ulcer in the angular incisure and **(B)** pyloric ring stenosis caused by a bleeding mucosal ulcer.

**Figure 2:**
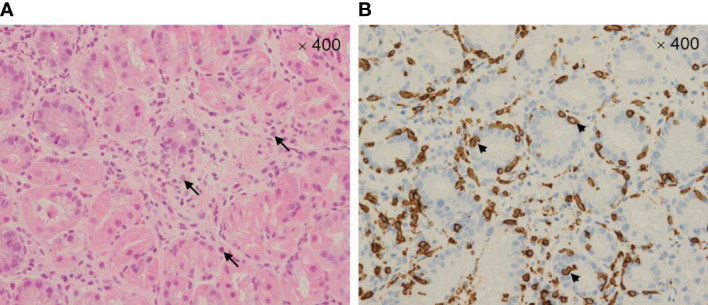
Microscopic image (400×) of the gastric mucosa stained with hematoxylin and eosin **(A)** showing marked intraepithelial lymphocytosis with prominent apoptotic bodies—arrows. Microscopic image (400×) of the immunohistochemically stained gastric mucosa **(B)** showing CD8^+^ T cell infiltrates—arrowheads.

**Figure 3:**
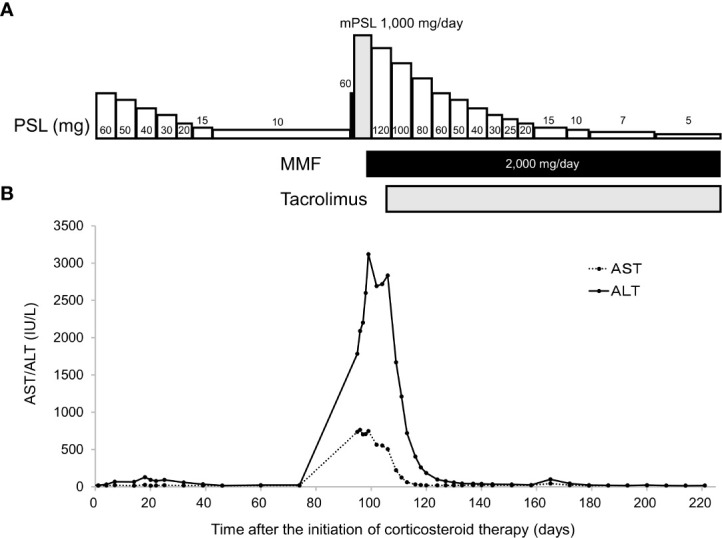
Time-course of immunosuppressant therapy **(A)**. Pulse corticosteroid therapy with mPSL 1,000 mg/day was started 97 days after initiating corticosteroid therapy with oral PSL. After 3 doses of the latter therapy, oral PSL 2 mg/kg/day was administered in combination with MMF. Time-course changes in serum AST and ALT levels **(B)**. Serum ALT levels increased despite co-treatment with PSL and MMF, which prompted the initiation of tacrolimus. Serum AST and ALT levels decreased after initiating tacrolimus administration. AST, aspartate aminotransferase; ALT, alanine aminotransferase; mPSL, methylprednisolone; PSL, prednisolone; MMF, mycophenolate mofetil.

A 58-year-old Japanese male, who was diagnosed with T4N2M0 p16-positive oropharyngeal SCC at the right lingual tonsis sulcus in the first computed tomography (CT), received cisplatin 80 mg/m^2^ IV every 3 weeks in 3 cycles and concurrent radiotherapy 70.4 Gy. SCC with multiple pulmonary nodular lesions recurred at 1 year and 2 months after completing the radiotherapy. Tumor analysis on the biopsy specimen collected at the initial diagnosis of oropharyngeal SCC revealed a PD-L1 combined positive score of 5. The patient was then treated with cisplatin 100 mg/m^2^ and pembrolizumab 200 mg, both on day 1 every 3 weeks for 5 cycles, as well as 5-fluorouracil 1,000 mg/m²/day on days 1 to 4 every 3 weeks for 5 cycles, followed by treatment with pembrolizumab 400 mg every 6 weeks. The patient complained of appetite loss and upper abdominal pain at 11 months after initiating treatment with pembrolizumab plus cisplatin and 5-fluorouracil. Upper gastrointestinal endoscopy (UGE) revealed a severe ulcer in the angular incisure and antrum of the stomach (**Figure 1A**), which was treated with esomeprazole 20 mg/day. Whole-body CT after the first UGE showed mural thickening of the stomach, not detected in whole-body CT before initiating treatment with pembrolizumab plus cisplatin and 5-fluorouracil, without imaging evidence of any tumor progression. At 12 months, the patient was admitted to our hospital with a history of progressive upper abdominal pain and a 5-kg weight loss over 14 days. After admission, the second UGE revealed exacerbated hemorrhagic gastritis, evenly spreading over the entire gastric mucosa and deep ulceration. The endoscope could not pass through the duodenum due to ulceration-induced stenosis of the pyloric ring (**Figure 1B**). Histopathology revealed lymphoplasmacytic infiltration into the fundic gland mucosa without evidence of infection or malignant neoplasia (**Figure 2A**). Immunohistochemical results indicated that most lymphocytes were CD8^+^ T cells (**Figure 2B**), suggesting pembrolizumab-induced gastritis. Therefore, pembrolizumab treatment was discontinued. After administering oral prednisolone 1 mg/kg, the symptoms gradually improved within a few weeks, and the dose was tapered over the subsequent 2 months. Follow-up UGE performed at 2 months later showed an improved ulcer and mucosal healing. Nevertheless, pyloric ring stenosis caused solid food dysphagia. During the course of maintenance therapy with prednisolone 10 mg/day for pembrolizumab-induced gastritis in the outpatient setting, the patient developed delayed severe hepatitis without deteriorating subjective symptoms and was readmitted to our hospital. The patient was diagnosed with “Grade 4 aspartate aminotransferase (AST) increase” and “Grade 4 alanine aminotransferase (ALT) increase”—AE grades defined in Common Terminology Criteria for Adverse Events version 5.0 (6). Whole-body CT and abdominal ultrasonography ruled out metastatic liver involvement; no sign of biliary tract disease was apparent. Viral causes of hepatitis (hepatitis A, B, and C viruses, cytomegalovirus, Epstein-Barr virus, herpes simplex virus, and varicella zoster virus) were excluded by serological tests. The patient was negative for the antinuclear antibody and antimitochondrial antibody. Although we did not conduct liver biopsy whose role in patients with suspected liver injury due to ICIs is controversial (7), the immunological origin of delayed severe hepatitis was strongly suspected based on the abovementioned test results and the development of delayed severe hepatitis during the treatment of pembrolizumab-induced gastritis. Hence, pulse corticosteroid therapy with intravenous methylprednisolone (1,000 mg/day) was administered for 3 days, followed by co-treatment with oral prednisolone (2 mg/kg/day) and oral mycophenolate mofetil (MMF) (2,000 mg/day) due to refractoriness to corticosteroid therapy. After 1 week of co-treatment with high-dose prednisolone and MMF, impaired liver function failed to improve substantially. Subsequently, oral tacrolimus, administered 8 days after the start date of MMF and reaching targeting serum trough concentrations of 8–10 ng/mL, gradually improved the AE grades of AST and ALT from “Grade 4” to “Grade 1.” The oral prednisolone dose was slowly tapered under close blood monitoring (**Figure 3**). The patient was discharged 60 days after hospitalization and had no indication of solid food dysphagia at 2 months after initiating tacrolimus treatment. Oral prednisolone and MMF were discontinued at 8 and 6 months of treatment, respectively. The oral tacrolimus dose was slowly tapered over 4 months, with treatment terminated 7 months after initiation. Follow-up UGE conducted at 7 months of immunosuppressant therapy with MMF and tacrolimus revealed improvement in pyloric ring stenosis, and the endoscope could pass through the pylorus. Treatment with pembrolizumab was not resumed. CT conducted at 10 months after discontinuing pembrolizumab revealed metastases to the mediastinal nodes and right hilar lymph nodes, as well as right pleural dissemination, although the primary tumor remained in remission. The patient preferred best supportive care despite chemotherapy with paclitaxel and cetuximab was proposed as the next treatment and died of cancer progression at 17 months after discontinuing pembrolizumab.

## Discussion

Herein, we report on a patient with pembrolizumab-induced gastritis followed by delayed severe hepatitis developed in a Japanese male. To the best of our knowledge, this is the first report on the concurrence of both inflammatory diseases. These inflammatory diseases were improved by adding tacrolimus to the co-treatment with prednisolone and MMF. Based on accumulated evidence, the incidence of ICI-induced colitis ranges from 8 to 27% (8), whereas ICI-induced gastritis occurs less frequently than irAEs in other organs (9). ICI-induced gastritis reportedly occurs at 4–9 months after initiating ICI treatment, with a later onset than ICI-induced colitis (10). Symptoms of ICI-induced gastritis include nausea, vomiting, abdominal pain, dyspepsia, anorexia, diarrhea, weight loss, dysphagia, and hematemesis (9). Our patient developed appetite loss and upper abdominal pain, which are not specific AEs of ICIs but are relatively common in patients with cancer. In ICI-induced gastritis, endoscopic findings include erythema, friability, denudation, edema, hemorrhage, granularity, erosions, ulcerations, and white exudates (9); however, these findings are common in other inflammatory diseases. Hence, ICI-induced gastritis can be difficult to diagnose based on endoscopic findings alone. ICI-induced gastritis manifests as diffuse chronic active gastritis, accompanied by increased intraepithelial lymphocytes and apoptosis (11). Considering the present patient, more than 1 month had elapsed after symptom onset before establishing a diagnosis of ICI-induced gastritis, as the possibility of irAEs was not considered. Consequently, the symptoms worsened despite proton pump inhibitor treatment. Therefore, clinicians should consider the possibility of ICI-induced gastritis when providing medical attention to patients receiving ICI therapy who present with gastrointestinal symptoms.

Hepatotoxicity, reported in 2–10% of patients treated with nivolumab, pembrolizumab, or ipilimumab monotherapy, predominately develops within the first 6–12 weeks of treatment (8). Our patient developed delayed severe hepatitis at 15 months after initiating pembrolizumab treatment, and “Grade 4 AST increased” and “Grade 4 ALT increased” occurred at 3 months after pembrolizumab treatment was discontinued. IrAEs occurring 90 days or later after discontinuation of immunotherapy have been deemed delayed irAEs (12). The present case report urges clinicians to pay heed to the potential development of new-onset hepatitis, even during maintenance corticosteroid therapy. The addition of tacrolimus to the co-treatment with prednisolone and MMF may be beneficial for steroid-refractory immune-related hepatitis (13). However, the therapeutic efficacy of tacrolimus added to the co-treatment with prednisolone and MMF for immune-related gastritis has not been reported. Although the gastritis-related symptoms experienced by our patient improved after initiating corticosteroid therapy, pyloric ring stenosis continued to cause dysphagia. However, the addition of tacrolimus to the co-treatment with prednisolone and MMF improved the stenosis. Steroid-refractory, nivolumab-induced esophageal stenosis completely resolved after personalized therapy with tocilizumab, an anti-interleukin-6 agent, suggesting a role for IL-6 blockade when managing severe steroid-refractory esophageal stenosis and more broadly refractory irAEs (14).

In conclusion, our patient who developed checkpoint inhibitor-induced gastritis followed by delayed severe hepatitis responded well to triple immunosuppressant therapy comprising prednisolone, MMF, and tacrolimus. Therefore, this immunotherapeutic approach could be effective for multiorgan irAEs in patients with cancer.

## Data availability statement

The original contributions presented in the study are included in the article/supplementary material. Further inquiries can be directed to the corresponding author.

## Ethics statement

Ethical review and approval was not required for the study on human participants in accordance with the local legislation and institutional requirements. The patients/participants provided their written informed consent to participate in this study. Written informed consent was obtained from the individual(s) for the publication of any potentially identifiable images or data included in this article.

## Author contributions

TO, YH, SM, and YI contributed to the conception and design of this manuscript. TO drafted the manuscript. TO, SM, YI, and YK contributed to the management of clinical cases and the interpretation of clinical data. TO, YH, MN and TK reviewed the manuscript. All authors contributed to the article and approved the submitted version.
